# Maternal Folate Excess, Placental Hormones, and Gestational Diabetes Mellitus: Findings from Prospective Cohorts Before and After Mandatory Folic Acid Food Fortification

**DOI:** 10.3390/nu17172863

**Published:** 2025-09-04

**Authors:** Tanja Jankovic-Karasoulos, Melanie D. Smith, Shalem Leemaqz, Murthy Mittinty, Jessica Williamson, Dylan McCullough, Anya L. Arthurs, Gustaaf A. Dekker, Claire T. Roberts

**Affiliations:** 1 Flinders Health and Medical Research Institute, College of Medicine and Public Health, Flinders University, Adelaide, SA 5042, Australia; melanie.smith@flinders.edu.au (M.D.S.); shalem.leemaqz@sa.gov.au (S.L.); murthy.mittinty@flinders.edu.au (M.M.); jessica.williamson@flinders.edu.au (J.W.); dylan.mccullough@flinders.edu.au (D.M.); anya.arthurs@adelaide.edu.au (A.L.A.); claire.roberts@flinders.edu.au (C.T.R.); 2 Robinson Research Institute, School of Biomedicine, The University of Adelaide, North Terrace, Adelaide, SA 5005, Australia; gustaaf.dekker@adelaide.edu.au; 3 Lyell McEwin Hospital, Haydown Road, Elizabeth Vale, SA 5112, Australia

**Keywords:** micronutrient supplementation, one-carbon metabolism, maternal metabolic health, hyperglycemia in pregnancy, pregnancy complications, placental endocrine function

## Abstract

**Background/Objectives:** Gestational diabetes mellitus (GDM) prevalence in Australia has increased from 5.6% (2010) to 19.3% (2022), coinciding with the introduction of mandatory folic acid (FA) food fortification and increased supplementation. Animal studies show that high FA intake in pregnancy impairs maternal glucose regulation, but the underlying mechanisms are unknown. We investigated whether fortification has altered maternal folate status to increase GDM risk, and whether key hormones that regulate maternal glucose homeostasis are affected following FA fortification. **Methods**: Serum folate, red cell folate (RCF), prolactin (PRL), human placental lactogen (hPL) and placental growth hormone (GH2) were measured in early pregnancy samples from women enrolled in prospective cohorts: SCOPE (N = 1164; pre-fortification) and STOP (N = 1300; post-fortification). Associations with GDM were assessed. **Results:** Compared to pre-fortification, women post-fortification had a higher GDM incidence (5.0% vs. 15.2%), serum folate (↑ 18%), RCF (↑ 259%), hPL (↑ 29%), and GH2 (↑ 13%) concentrations. RCF concentrations above the clinical reference range were found in 57.6% of women post-fortification. Causal mediation analysis suggests that higher RCF contributed to increased GDM risk. Women with RCF excess had 48% more GDM cases, and higher PRL (↑ 24.2%) and hPL (↑ 12.7%) levels compared to those within the reference range. **Conclusions**: Maternal folate excess is likely contributing to the rising prevalence of GDM in Australia. These findings highlight the need to evaluate excess FA/folate safety in pregnancy, particularly in countries with mandatory fortification. Placental hormones may represent a mechanistic link between excess folate and GDM, warranting further investigation.

## 1. Introduction

**Figure 1:**
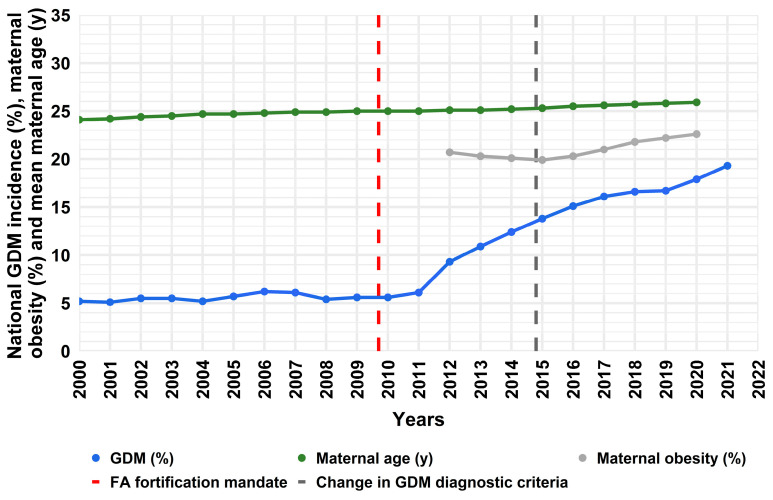
Incidence of GDM, maternal age and obesity in Australia (AIHW trends over time data) [1]. National GDM incidence is indicated by the solid blue line; Mean national maternal age is shown in green; National maternal obesity data (gray) is incomplete prior to 2012; Dashed vertical lines indicate implementation of FA food fortification mandate (red) and the change in WHO diagnostic criteria (dark gray).

According to national surveillance data, gestational diabetes mellitus (GDM) incidence has more than tripled in Australia in just over a decade (Figure 1) [1]. The most recent change in GDM diagnostic criteria by the World Health Organization (WHO) [2] was implemented in Australia in 2015. While the diagnostic change has often been cited to account for the rising GDM rates, the steep increase in incidence both predates its introduction and continues thereafter (Figure 1). Well established GDM risk factors such as maternal obesity and age (Figure 1) as well as ethnicity, have marginally changed during the same period. Although these factors may have contributed toward the GDM rise, even combined they cannot explain the observed trajectory. Moreover, the same risk factors also increase the risk for hypertensive disorders of pregnancy which are not on the same trajectory as GDM. This suggests that additional factors are involved. Observational studies link high folic acid (FA; synthetic folate) intake with increased GDM risk [3,4,5,6], while animal studies provide compelling evidence that high FA intake during pregnancy plays a role in insulin resistance and impaired glucose handling [7,8,9,10], but the mechanisms remain unknown.

The WHO recommends women supplement with 400 µg FA daily when trying to conceive until 12 weeks’ gestation, to reduce the risk of fetal neural tube defects [11]. Accordingly, FA supplementation with a daily dose of 400 µg has been part of the Australian clinical pregnancy guidelines for more than 20 years. However, given the neural tube closes ~4 weeks post-conception [12], before most women know they are pregnant, and that many pregnancies are unplanned, in 2009 the government mandated FA fortification of flour for breadmaking purposes. Concurrent with this has been an increase in the use of FA-containing prenatal multivitamins, with some leading brands containing double the recommended FA dose (800 μg). Our recent study shows that the period spanning FA fortification mandate was accompanied by an increase in the use of supplements that contain 800 µg or more FA, from 24% pre fortification to over 60% post fortification [13].

A recent systematic review of women taking FA supplements in countries with mandatory food fortification programs, including Australia, found that almost all women exceed 1000 μg daily limit [14]. Whilst this limit is set due to potential masking of B12 deficiency, to date there has been no established upper limit for adverse pregnancy outcomes. Importantly, recent studies report an increase in women who continue to take FA-containing multivitamins throughout pregnancy [15], despite no known benefit or established harm from continuing to supplement beyond 12 weeks, long after the neural tube has closed. Increased dose and duration of FA supplementation, and high levels of unmetabolized FA and folate in maternal blood during pregnancy, have raised concerns about potential harm as highlighted in a systematic review by Xu et al. (2023) [16] and a narrative review by Williamson et al. (2022) [6].

To determine whether increased FA intake over the past decade has placed Australian women at increased risk of GDM, we leveraged two large prospective pregnancy cohorts that were recruited at the same hospital prior to (SCOPE), and post (STOP), the 2009 FA fortification mandate. To our knowledge, no previous study has examined the impact of population-level fortification policy on maternal folate status and GDM incidence using two comparable cohorts recruited at the same hospital before and after fortification, providing a unique opportunity to minimize variability related to geography or healthcare setting. We therefore set out to determine the incidence of GDM in these cohorts to ensure that our local data reflects the broader population trend, and to measure maternal folate status to establish risk. Furthermore, in search of a mechanism by which FA may influence GDM risk, we turned to the placenta, an organ unique to pregnancy that plays a central role in regulating maternal glucose homeostasis. The placenta secretes hormones into the maternal circulation such as placental growth hormone variant (GH2) and human placental lactogen (hPL) which, along with prolactin (PRL) released from the maternal pituitary and uterine decidua, modulate maternal insulin sensitivity and glucose homeostasis [17,18]. These hormones contribute to the physiological insulin resistance of pregnancy, a critical adaptation that increases maternal glucose availability for the fetus, while also promoting compensatory insulin secretion to maintain maternal glucose homeostasis and prevent hyperglycaemia and GDM [17].

Our hypothesis that FA may exert its effects via the placenta is further supported by epidemiological data: while GDM incidence has increased markedly, the prevalence of Type 2 diabetes—despite its pathophysiological similarities to GDM—has shown no comparable increase in Australia during the same period [19]. This divergence suggests that FA may exert pregnancy-specific effects on glucose metabolism.

Given that placental function is highly reliant on maternal nutrient supply [20], and that FA/folates play a key role in epigenetic regulation of gene expression and protein synthesis [21], it is plausible that increased FA/folate supply from the mother to the placenta can alter placental function (hormone secretion) placing women at increased risk of GDM. We aimed to determine whether high maternal folate increases the risk for GDM in women post FA fortification, and whether placental hormones that regulate maternal glucose homeostasis are involved. We hypothesized that increased FA intake following fortification will substantially elevate maternal folate status, thereby increasing the risk of GDM, and that concentrations of placental hormones involved in maternal glucose regulation will be altered in women exposed to high folate levels post-fortification. To our knowledge, this is the first study to examine maternal folate levels in relation to both GDM risk and placental hormone profiles across pre- and post-fortification cohorts, providing novel insights into the associations between chronic FA exposure, pregnancy-specific endocrine function and GDM risk.

## 2. Materials and Methods

### 2.1. Study Cohorts and Criteria

We used data from two prospective pregnancy cohorts that recruited women at the Lyell McEwin Hospital, Adelaide, South Australia. The Adelaide branch of the international SCOPE (Screening for Pregnancy Endpoints) cohort recruited nulliparous women with singleton pregnancies prior to the FA fortification mandate (2005–2008; Total N = 1164), while the STOP (Screening Tests to identify poor Outcomes of Pregnancy) cohort was recruited 6–9 years post fortification mandate (2015–2018; Total N = 1300). Women at high risk of pregnancy complications due to underlying medical history (e.g., type 1 or type 2 diabetes, hypertension, and related disorders), 3 or more previous miscarriages or terminations of pregnancy, or those whose pregnancy was complicated by a known fetal anomaly or if they received interventions that may modify pregnancy outcome (e.g., aspirin) were excluded from both studies. Women were also excluded if they were taking calcium (>1 g/d), eicosapentaenoic acid (≥2.7 g/d), vitamin C (>1000 mg/d) or vitamin E (>400 IU/d). Only women with a confirmed GDM pregnancy outcome were included in data analyses. Importantly, SCOPE and STOP GDM incidence was calculated using the same WHO diagnostic criteria [2]. Non-GDM cases include uncomplicated pregnancies as well as pregnancies affected by pathologies such as Gestational hypertension (GHTN), Preeclampsia (PE), Spontaneous Preterm Birth (sPTB) and Small for Gestational Age (SGA). SCOPE and STOP study ethics approvals were obtained from the Central Northern Adelaide Health Service Human Research Ethics Committee (REC1712/5/2008) and the Women’s and Children’s Health Network Human Research Ethics Committee (HREC/14/WCHN/90), respectively. SCOPE and STOP were registered with the Australian New Zealand Clinical Trial Registry (ACTRN12607000551493 and ACTRN12614000985684, respectively).

### 2.2. Blood Biochemical Measurements

**Figure 2:**
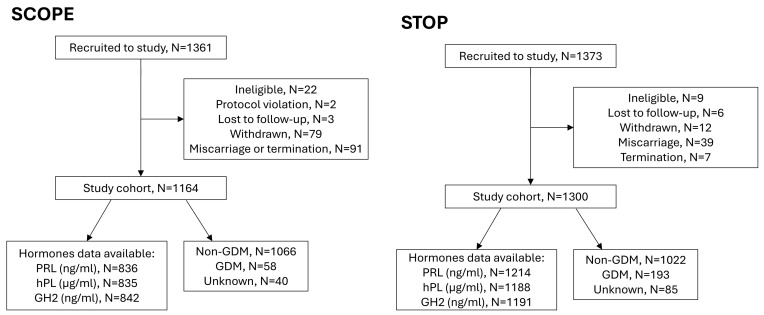
Flow diagram for SCOPE and STOP studies showing final sample numbers available for hormone measurements.

Peripheral non-fasting blood samples collected at 14–16 (SCOPE; Mean gestational age 15 weeks’) and 6–16 (STOP; Mean gestational age 12 weeks’) weeks of gestation were placed on ice, processed, and stored at −80 °C for future analyses. Serum folate, red cell folate (RCF), PRL, hPL and GH2 were measured as described previously [13]. Sample numbers available for each hormone analysis are presented in Figure 2. RCF measurements were available for 1191 women in the post FA fortification STOP cohort but not for SCOPE. However, we previously published RCF data from 410 women at 12 weeks’ gestation who were recruited at the same hospital and at the same time as SCOPE women, and thus prior to FA fortification mandate [13]. This enabled us to compare changes in RCF in pregnancies pre and post FA fortification mandate.

### 2.3. Statistical Methodology

Descriptive statistics for maternal characteristics are reported by cohort. We conducted an individual participant data meta-analysis to allow for the case-mix heterogeneity (arising when the intervention effect is modified by one or more of the confounders used in defining the case-mix) and “beyond case-mix heterogeneity” (due to study differences based on design or methodological aspects). Following the work of Vo et al. (2019) [22], we performed a meta-analysis involving two cohorts (SCOPE and STOP) to compare the effect of serum folate concentration as continuous exposure on a dichotomous outcome GDM. As each participant has only one observed outcome, a counterfactual outcome (if the participant was assigned to a different folate exposure) is calculated. To adjust for observed confounding, inverse probability treatment weights (IPTW) were obtained for the continuous exposure (serum and red cell folate concentrations) with maternal BMI, age, ethnicity, SEI, and metabolic syndrome as covariates within each study, where a normal probability density is assumed. Due to other epidemiological factors that may induce differences between the cohorts, the version of folate exposure is likely to be different between cohorts, hence a propensity of study was estimated using Logistic regression with the same covariates. An overall weight was obtained by multiplying the treatment weights with the propensity of study. To estimate relative risk and corresponding 95% confidence intervals we used log-binomial regression to fit the outcome model, with serum folate as the exposure and GDM as the outcome, weighted by the overall weight, we also used study as confounder. The IPTW analysis was conducted under the assumptions of consistency, positivity, and ignorable treatment (Appendix A). To analyze the trend of hormone concentration across gestation, linear model with cluster-robust Sandwich variance was used. This allows for prediction of the mean hormone level at 16 weeks’ gestation, adjusted for the gestational age at sampling. Hormone levels were log-transformed to approximate normality, and cohort, gestational age, and their interaction term included as predictors. Causal mediation analysis (which allows inference of causation) was performed to estimate the direct effect of red cell folate on the odds of GDM and the joint indirect effect of red cell folate on GDM through hPL and GH2 (Appendix B). The natural direct and indirect effects were obtained using the imputation approach [23] and medflex R package (version 4.4.2) [24]. All statistical analyses were performed using R version 4.3.2 (R Foundation for Statistical Computing, Vienna, Austria).

## 3. Results

### 3.1. Comparative Assessment of Maternal Factors and Pregnancy Outcomes in SCOPE and STOP

Maternal characteristics and pregnancy outcome data for 1164 SCOPE, and 1300 STOP pregnancies are presented in Table 1. STOP data are compared to SCOPE throughout this section.

STOP women were older, had a higher BMI, SEI (but still disadvantaged), were less likely to be Caucasian and less likely to smoke cigarettes (Table 1).

At least 60% of STOP women reported daily FA supplementation of 800 μg or more, compared to 25.9% in SCOPE (Table 1). The magnitude of this difference is likely an underestimation due to 11.7% missing supplementation data in STOP. Folic acid supplementation categories were based on the recommended daily supplementation dose in pregnancy (400 μg) and the commonly used higher dose of 800 μg found in many prenatal supplements.

Fewer STOP women met the criteria for metabolic syndrome, although some data were missing (7.1%; Table 1). Importantly, even if all missing cases were positive, the incidence of metabolic syndrome would remain lower than in SCOPE. This is important given that metabolic syndrome is an independent risk factor for GDM [25].

All assessed pregnancy outcomes were comparable between cohorts, except for GDM (Table 1), which showed a marked increase in the STOP cohort (Table 1). The incidence of GDM had more than tripled, aligning with the national rise over the same period (Figure 1). Importantly, this increase remained significant after adjusting for established risk factors including maternal age, BMI, ethnicity, SEI and metabolic syndrome.

### 3.2. Maternal Folate Status Pre and Post FA Fortification

Serum folate concentrations reflect recent intake and must be interpreted with caution in non-fasting samples. Nevertheless, compared to women pre-FA fortification, women post FA fortification had 18% higher serum folate (Median [IQ range]: 36 [28.2–2.8] vs. 40.5 [36.3–45.3] nmol/L, *p* < 0.0001).

**Figure 3:**
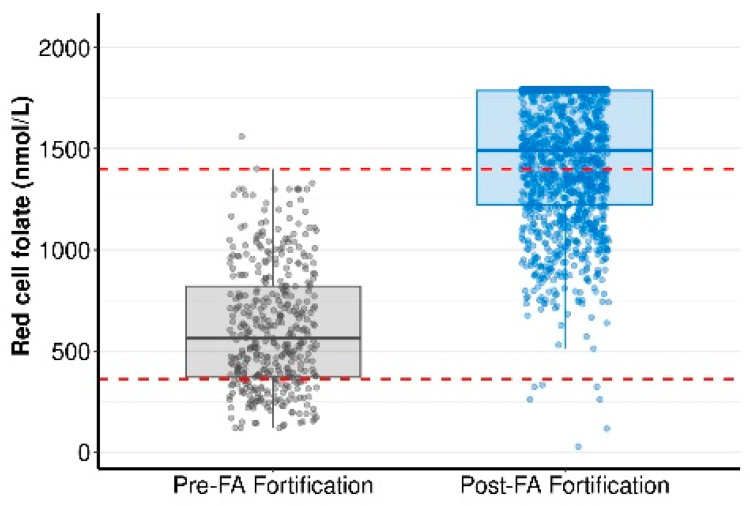
Red cell folate (RCF) concentration in pregnant women prior to and post FA food fortification. Data in the pre- and post- FA fortification categories are presented as median (IQ range). Red dotted lines indicate the normal reference range for RCF (360–1400 nmol/L) set by the Royal College of Pathologists of Australasia (RCPA). Assay upper limit of detection is 1790 nmol/L. RCF measures were compared between STOP (post-FA fortification; using N = 1191 samples for which we had available RCF data) and a separate pre-FA fortification cohort (not SCOPE, N = 410) using Mann–Whitney U test.

Although The Royal College of Pathologists Australasia (RCPA) recommends measuring serum folate for diagnosis of folate deficiency, red cell folate (RCF) concentration is a more reliable indicator of long-term folate stores and importantly it is not significantly affected by whether the blood sample is taken in a fasting or non-fasting state. Furthermore, RCF concentration is a surrogate marker of tissue folate levels [26]. Compared to women pre-FA fortification, RCF was 259% higher in women post FA fortification (Median [IQ range]: 561 [370–820.5] vs. 1490 [1222.5–1787.5] nmol/L, *p* < 0.0001). This difference is likely underestimated as a large proportion of post FA fortification samples reached the upper detection limit of the assay (1790 nmol/L; Figure 3).

The *normal* reference range for red cell folate is 360–1400 nmol/L (indicated by the red lines in Figure 3). In this study, RCF levels below this range are referred to as *deficient*, levels above the reference range but below the assay’s upper detection limit (1790 nmol/L) as *elevated*, and levels at or potentially above the detection limit as *excess*. The proportion of women with RCF deficiency declined from 22.7% (N = 93) pre-FA fortification to just 0.6% (N = 7) post FA fortification (Figure 3). In contrast, the proportion of women with RCF levels above the normal reference range increased markedly from 0.5% (N = 2) pre-FA fortification to 57.6% (N = 686) post FA fortification (Figure 3). These findings suggest that, in the post fortification era, folate excess has become far more common than deficiency.

### 3.3. Red Cell Folate Increases GDM Risk in Women Post-Fortification

An inverse probability weighting model was used to assess the overall relative risk of developing GDM in STOP compared to SCOPE. After adjusting for established GDM risk factors (maternal age, BMI, ethnicity, SEI, and metabolic syndrome), serum folate alone did not significantly increase GDM risk. However, a combination of serum folate and study (i.e., post-fortification) placed women post FA fortification at greater than three-fold increased risk of developing GDM compared to women pre- FA fortification (Table 2). This risk estimate is consistent with the measured three-fold increased GDM incidence in STOP compared to SCOPE (Table 1).

As RCF data were not available for SCOPE, we could not directly assess whether the 259% increase in RCF post FA fortification accounted for the cohort effect on GDM risk. However, when we analyzed the STOP data alone, to determine what factors increased GDM risk *within* the cohort, we found that both triglycerides and RCF significantly increased the relative risk of GDM in STOP women after adjusting data for known GDM risk factors including maternal age, BMI, ethnicity, SEI, and metabolic syndrome. For every 0.5 unit increase in triglycerides the risk of developing GDM increased by 28% (Table 3). Whilst triglycerides are an independent GDM risk factor in STOP, they are unlikely to explain the increased GDM incidence in STOP compared to SCOPE as a similar relative risk was observed in SCOPE (Relative risk for 0.5 unit increase in triglycerides in SCOPE [95% CI]: 1.34 [1.2–1.5], *p* < 0.0001), and median triglyceride levels were lower in STOP compared to SCOPE (Median [IQR] 1.1 [0.9–1.4] mmol/L vs. 1.4 [1.1–1.8] mmol/L, *p* < 0.0001, respectively).

In contrast, changes in RCF could explain the increase in GDM incidence in STOP. For every 500 nmol/L increase in RCF the risk of developing GDM increased by 34% (Table 3). Given the rise in median RCF from 561 [370–820.5] nmol/L pre- FA fortification to 1490 nmol/L post FA fortification (Median [IQ range]; *p* < 0.0001; Figure 3), this substantial increase in folate status may plausibly contribute to increased GDM risk and higher GDM incidence in STOP compared to SCOPE.

### 3.4. Red Cell Folate Strata Reflect a Stepwise Rise in GDM Incidence in Women Post Fortification

To support the risk prediction findings that higher RCF increases GDM risk in women post FA fortification, we stratified the measured GDM incidence in STOP by red cell folate status (using RCF data presented in Figure 3). Findings are presented in Table 4.

GDM prevalence increased progressively across RCF strata in the STOP cohort: from 12.5% among women with normal RCF to 16.2% among women with elevated RCF, and 18.5% among women with excess RCF concentrations (Table 4). Compared to women whose RCF was within the reference range, those with RCF excess had 48% more GDM cases (*p* = 0.03).

Given that one GDM case was observed among only seven women with folate deficiency, the small sample size limits interpretation of this subgroup.

### 3.5. Placenta but Not Pituitary Secreted Hormone Concentrations Are Altered Post FA Fortification

Numerous hormones regulate maternal insulin resistance and insulin secretion during pregnancy. We investigated whether some of these hormones were altered in women post FA fortification.

Because pregnancy hormone concentrations can fluctuate across gestation [17], we accounted for gestational age at sampling using a linear model with cluster-robust sandwich variance, based on repeated blood sample measures from the same SCOPE participants at 11–13 and 14–16 weeks’ (N = 22). This approach enabled estimation of marginal mean hormone concentrations at 16 weeks’ for both SCOPE and STOP cohorts—a time point that marks the beginning of the exponential rise in hormones and represents the latest sampling time in SCOPE.

*Pituitary* secreted PRL was not different between SCOPE and STOP (Table 5). However, compared to SCOPE, *placental* secreted hPL and GH2 concentrations were 25% and 13% higher in STOP (Table 5).

Interestingly, hPL as well as PRL concentrations were significantly higher in STOP women with RCF excess compared to those within the normal range—hPL was 12.8% higher (Median [IQ range]: 56.6 [37.2–87.1] vs. 63.8 [44.6–97]; *p* = 0.02) and PRL was 24.2% higher (Median [IQ range]: 71.7 [47.4–111.3] vs. 88.3 [55.1–133.5]; *p* = 0.005).

### 3.6. Folate May Act via Placental Hormones to Increase Risk of GDM

We analyzed whether the effect of RCF on GDM involved placental hormones GH2 and hPL. The causal mediation analysis described by Samoilenko et al. (2023) [27] was applied. Data were adjusted for maternal age, BMI, ethnicity, SEI, metabolic syndrome and gestational age at sampling. For every 500 nmol/L increase in RCF, GH2 increased and hPL decreased the risk of GDM, which is in line with their established roles in promoting insulin resistance and secretion, respectively. However, given that the two hormones do not act in isolation we implemented the joint mediation analysis described by Vansteelandt et al. (2012) [23] (Appendix B). There is not enough evidence from this study to show significance of an indirect effect, mediated by GH2 and hPL in combination, on GDM risk (RR [95% CI]: 1.00 [0.99, 1.02]).

## 4. Discussion

### 4.1. Increased GDM Incidence Post-Fortification

Our pregnancy cohort data indicate an alarming tripling in GDM incidence, consistent with the Australian national GDM trajectory [1]. Women in both of our pregnancy cohorts were screened for GDM and, importantly, its incidence was calculated using the same WHO diagnostic threshold. It is noteworthy that changes in GDM diagnostic threshold account for an increase in GDM incidence from 4.4 to 5% in SCOPE. This suggests that the change in diagnostic criteria that was implemented in 2015 across Australia is likely to explain some of the increase in national GDM incidence but not the magnitude of the increase nor the steep trajectory. Our study also suggests that known GDM risk factors, such as maternal obesity, age and ethnicity combined cannot completely explain the magnitude of the increase in GDM between the cohorts, as after adjusting data for multiple known confounders, GDM incidence was significantly different between cohorts. Furthermore, other pregnancy complications associated with the same risk factors were not higher in STOP (nor are they rising to the same degree nationally). This suggests that other factors may be contributing to the rising GDM incidence in Australia.

### 4.2. Maternal Folate Excess: A Potential Contributor to the GDM Rise in Australia

A constellation of known and novel factors has likely contributed to increased national GDM incidence. Based on the data presented herein and supported by evidence from clinical [3,4,5,6] and animal studies [7,8,9,10], we propose that elevated maternal folate status may be a contributing factor to the rising incidence of GDM in Australia. A combination of FA food fortification, both mandatory and voluntary, increased dose of supplementation and continued supplementation beyond the recommended first trimester, have resulted in maternal folate excess, levels that far exceed the established clinical reference range. Previous studies have reported a U-shaped relationship between the dose and duration of FA supplementation and GDM risk [4,28], and from that perspective excess maternal folate may biologically mimic folate deficiency, as previously reported for other biological measures. Although numbers in the deficient RCF category were low in our cohort, our observations also suggest a potential U-shaped relationship between maternal RCF levels and GDM incidence. Given that detrimental effects of maternal folate deficiency (characterized using similar reference range) on pregnancy health are well documented [20,29], the lack of evidence for the safety of excess maternal folate is concerning, particularly as folate excess is now far more common than folate deficiency. A recent NIH workshop highlighted the need for comprehensive research to bridge the knowledge gaps in understanding the metabolic and clinical effects of excess folates/FA [30]. Addressing this need, we show that increased RCF levels in women post FA fortification significantly increased GDM risk and that women with RCF excess had the highest proportion of GDM. These findings support previous reports of an association between increasing RCF during pregnancy and increased GDM risk [31,32].

A likely explanation for the observed association between RCF and GDM risk, but the absence of a similar association with serum folate, is the use of non-fasting blood samples in this study. Serum folate reflects recent folate/FA intake and is subject to short-term fluctuations influenced by meal timing and supplement use [33]. In contrast, RCF provides a more stable measure of long-term folate status [26], making it a more reliable marker when assessing associations between non-fasting blood samples with pregnancy outcomes such as GDM.

### 4.3. Maternal Folate Excess Is Associated with Altered Placental Hormone Levels

Despite convincing evidence from observational and animal studies for the role of FA in GDM etiology, to date the mechanisms remain unknown. Our study shows that women with folate excess had higher hPL and PRL (both primarily promote insulin secretion, although hPL has also been reported to promote insulin resistance) compared to women whose folate was within the normal range. Whilst it is possible that higher hPL and PRL levels in women with folate excess may indicate increased glucose tolerance, based on previous work they are more likely to reflect reduced glucose tolerance [34]. Whatever the biological relevance of these hormones may be as it relates to hyperglycaemia, the alteration of these hormones in folate excess warrants further study.

Importantly, our study shows that pregnant women post FA fortification had increased placenta-secreted (hPL and GH2) but not maternal-secreted (PRL) hormones in early gestation. Due to the established roles of these hormones in regulation of maternal glucose homeostasis, this finding supports our hypothesis that the placenta may be involved. Whilst the exact mechanism by which hPL affects glucose homeostasis remains unknown, GH2 has been shown to trigger severe insulin resistance in an animal model, suggesting that increased levels of GH2 post-fortification may place women at increased risk of insulin resistance [35]. Our mediation analyses indicated significant indirect effects of maternal folate on GDM risk via each placental hormone individually: GH2 increased risk, whereas hPL reduced risk, findings that are consistent with their established physiological roles in promoting maternal insulin resistance and insulin secretion, respectively [17,18,35]. However, when both hormones were modeled together, the combined indirect effect was not statistically significant. This likely reflects the opposing directions of effect, the limited sample size of this cross-sectional study, and the possibility that other placental hormones also contribute to the glucose regulation. We therefore interpret these findings as exploratory, providing the first evidence in human cohorts that placental endocrine function may partially mediate the relationship between maternal folate status and GDM risk. While not definitive, these results highlight a biologically plausible mechanism that warrants confirmation in larger studies with more comprehensive endocrine profiling.

### 4.4. Clinical Implications

Our findings may have several important clinical implications. First, the tripling in GDM incidence within less than a decade, which reflects the national trend, does not appear to be fully explained by known risk factors or by changes in diagnostic criteria, as is often assumed. Given the short- and long-term adverse consequences of GDM for both mother and child, there is a critical need to clarify the underlying drivers of the rising rates in Australia. Importantly, these observations may have broader relevance for other countries experiencing similar increases. Second, the success of mandatory FA fortification in preventing maternal folate deficiency has been offset by growing evidence of maternal folate excess, with unknown consequences for pregnancy health. The associations between excess FA/folate and GDM risk observed in our study and others, together with animal evidence that excessive FA intake can impair glucose homeostasis, highlight the need to consider potential unintended consequences of current supplementation and fortification policies. Third, our results suggest that defining an upper safe limit for maternal FA intake and folate status during pregnancy may be warranted. Fourth, the risk–benefit balance of supplementation may need re-evaluation: while early pregnancy supplementation remains essential for neural tube defect prevention, ongoing high-dose supplementation in the context of widespread fortification may provide limited additional benefit and could pose risks. Therefore, in the context of widespread fortification, a universal approach to supplementation may be less appropriate than more personalized strategies, such as assessing maternal folate status before recommending supplementation. Finally, our observation that placental hormone secretion is altered in women post-fortification and those with folate excess raises the possibility of a mechanistic pathway through which FA exposure could influence maternal insulin resistance and GDM risk. Collectively, these findings support the need to examine the combined effects of FA fortification and supplementation within the real-world setting of widespread multivitamin use, with the goal of preserving fetal neuroprotection while minimizing unintended metabolic risks for mothers and their babies.

### 4.5. Study Strengths and Limitations

We acknowledge that the two cohorts (SCOPE and STOP) were collected nearly a decade apart, and that demographic or contextual shifts over time may introduce residual confounding. However, both cohorts were recruited at the same hospital, serving women from the same catchment area, and were subject to consistent inclusion criteria and study protocols. Moreover, our analyses were adjusted for maternal age, BMI, ethnicity, socio-economic status, and metabolic syndrome. While residual confounding cannot be fully excluded, the magnitude and consistency of the observed increase in GDM incidence and maternal folate status post-fortification suggest that temporal demographic changes alone are unlikely to account for these findings.

We further acknowledge that pre-fortification RCF values were not available for the SCOPE cohort itself, but were derived from a separate dataset collected at the same time and at the same hospital, using identical protocols and assays. While this does not provide a perfectly matched comparison, the large magnitude of difference observed between pre- and post-fortification RCF levels makes it unlikely that methodological variability alone accounts for our findings. Residual confounding from unmeasured temporal or societal changes cannot be excluded, and our results should be interpreted with caution.

Lastly, we also acknowledge limitations in the use of inverse probability of treatment weighting (IPTW). As with all causal inference approaches in observational studies, the assumptions of ignorability and positivity cannot be fully verified, particularly when cohorts are separated by time. While IPTW was applied to both serum folate and RCF data to adjust for key confounders, the strongest evidence in our study arises from the within-STOP IPTW analysis of RCF, which avoids temporal cohort comparisons. Importantly, the consistency of associations across weighted and unweighted analyses, as well as the stepwise increase in GDM incidence across RCF strata, supports the robustness of our conclusions despite these limitations.

### 4.6. Future Directions

Studies are needed to establish an upper safety threshold for both FA intake and maternal folate status during pregnancy. In addition, the role of the placenta in mediating the effects of FA on GDM warrants further exploration, not only through its regulation of endocrine peptides known to regulate maternal glucose homeostasis (including leptin, estradiol, progesterone, etc.), but also via other placental non-endocrine functions which may contribute to metabolic adaptations in pregnancy. Other than our recently published study which showed alterations in placental hormones in women with uncomplicated pregnancies following FA fortification [13], we are unaware of other studies that have investigated the relationship between FA/folate and placental hormones in the context of insulin resistance, hyperglycemia and/or GDM. Finally, further research is needed to determine why some women may be more susceptible than others to the metabolic effects of FA, which could provide new insights into the etiology of GDM.

## 5. Conclusions

Our study confirms a significant increase in GDM incidence in women post FA fortification. While residual confounding cannot be excluded, our findings suggest that maternal folate excess may be contributing to the rising prevalence of GDM in Australia. We also observed that women post-fortification as well as those with excess folate, have altered levels of placental hormones that play a role in the regulation of maternal glucose homeostasis. Further investigation is warranted to establish the role of FA in the regulation of placental endocrine function, particularly in the context of GDM. Assessment of the safety of maternal folate excess in pregnancy is required. Evaluations of efficacy and safety of the current real world FA supplementation practice during pregnancy in the era of FA food fortification are warranted. Improved guidelines on FA supplementation during pregnancy would protect the fetus in early gestation against NTDs but also protect the mother and fetus from adverse effects of hyperglycemia. Given the duration and extent of FA supplementation in pregnancy is highly modifiable, understanding potential harms of excess FA intake is of major public health importance.

## Figures and Tables

**Table 1:** Maternal characteristics assessed in early gestation in the SCOPE (15 weeks’) and STOP (12 weeks’) cohorts with available term pregnancy outcome data.

	SCOPE (N = 1164)	STOP (N = 1300)	*p*-Value
Maternal age (y): Median (IQ range)	23.0 (20.0–27.0)	26.0 (22.0–29.0)	<0.0001
Maternal BMI: Median (IQ range)	25.6 (22.2–30.8)	26.3 (22.7–31.8)	0.006
Maternal BMI (category): N(%)			0.02
Missing	0 (0.0)	1 (0.1)	
<20	122 (10.5)	95 (7.3)	
≥20 to <25	398 (34.2)	442 (34.0)	
≥25 to <30	322 (27.7)	361 (27.8)	
≥30 to <40	267 (22.9)	315 (24.2)	
≥40	55 (4.7)	86 (6.6)	
SEI *: Median (IQ range)	25.0 (20.0–30.0)	29.0 (22.0–45.0)	<0.0001
Ethnicity: Caucasian N(%)			0.0005
Yes	1067 (91.7)	1073 (82.5)	
No	97 (8.3)	227 (17.5)	
Smoking: N(%)			<0.0001
Missing	0 (0.0)	7 (0.5)	
No	886 (76.1)	1077 (82.8)	
Yes	278 (23.9)	216 (16.6)	
Folic acid supplementation (μg/day): N(%)			<0.0001
Missing	0 (0.0)	152 (11.7)	
None	251 (21.6)	97 (7.5)	
≤400	116 (10.0)	15 (1.2)	
>400 to <800	495 (42.5)	256 (19.7)	
≥800	302 (25.9)	780 (60.0)	
Metabolic Syndrome: N(%)			<0.0001
Missing	11 (0.9)	92 (7.1)	
No	953 (81.9)	1096 (84.3)	
Yes	200 (17.2)	112 (8.6)	
Pregnancy Outcome			
Gestational diabetes mellitus (GDM) ^†^	58 (5.0)	198 (15.2)	<0.0001
Gestational hypertension (GHTN)	94 (8.1)	83 (6.4)	0.14
Preeclampsia (PE)	117 (10.1)	120 (9.2)	0.37
Spontaneous preterm birth (sPTB)	69 (5.9)	61 (4.7)	0.14
Small for gestational age (SGA) ^‡^	141 (12.1)	155 (11.9)	0.65

* SEI = New Zealand Socio-economic Index, a scale of 10–90 with a lower score indicating greater disadvantage; ^†^ SCOPE and STOP GDM incidence were calculated using the same 2014 diagnostic criteria (old criteria applied at the time of SCOPE (2005−2008) had SCOPE GDM incidence at 4.4%); ^‡^ SGA defined as birthweight below the 10th customized centile adjusted for maternal height, weight, parity, ethnicity, gestational age at delivery and infant sex.

**Table 2:** The effect of serum folate and study (STOP vs. SCOPE) on GDM risk.

	Relative Risk (95% CI)	*p*
Effect of serum folate without study		
Per 10-unit increase	1.02 (0.98, 1.05)	0.3
Per 50-unit increase	1.09 (0.93, 1.27)	0.3
Effect of serum folate with study		
Per 10-unit increase	3.12 (2.26, 4.29)	<0.0001
Per 50-unit increase	3.12 (2.20, 4.42)	<0.0001

Data adjusted for maternal age, BMI, ethnicity, SEI, and metabolic syndrome.

**Table 3:** Factors contributing to GDM risk in women post FA fortification (STOP).

	Relative Risk (95% CI)	*p*
Effect of triglycerides		
Per 0.1 unit increase	1.05 (1.03, 1.07)	<0.0001
Per 0.5-unit increase	1.28 (1.14, 1.43)	<0.0001
Effect of red cell folate (RCF)		
Per 100-unit increase	1.06 (1.00, 1.12)	0.04
Per 500-unit increase	1.34 (1.01, 1.78)	0.04

Data adjusted for maternal age, BMI, ethnicity, SEI, and metabolic syndrome.

**Table 4:** GDM incidence stratified by RCF category among 1191 STOP women with available RCF data.

RCF Reference Range	Total Number of Women (N)	Proportion of Women with GDM—N (%)
<360 nmol/L (deficient)	7	1 (**14.3**)
360–1400 nmol/L (normal)	498	62 (**12.5**)
>1400–<1790 nmol/L (elevated)	389	63 (**16.2**)
≥1790 nmol/L (excess)	297	55 (**18.5**)

**Table 5:** Hormone concentrations, presented as Estimated Marginal Means (SEM), at 16 weeks’ gestation for SCOPE and STOP women.

**Estimated Marginal Means (95% CI)**	**SCOPE**	**STOP**	***p***
PRL (ng/mL)	157.7 (148.7, 167.2)	145.8 (126.8, 167.65)	0.3
hPL (µg/mL)	191.8 (187.2, 196.5)	239.8 (225.0, 255.5)	<0.0001
GH2 (ng/mL)	4.7 (4.5, 4.8)	5.3 (4.9, 5.7)	0.008

SCOPE and STOP sample numbers used for each analysis are shown in Figure 2. Data is adjusted for gestational age at sampling.

**Figure A1:**
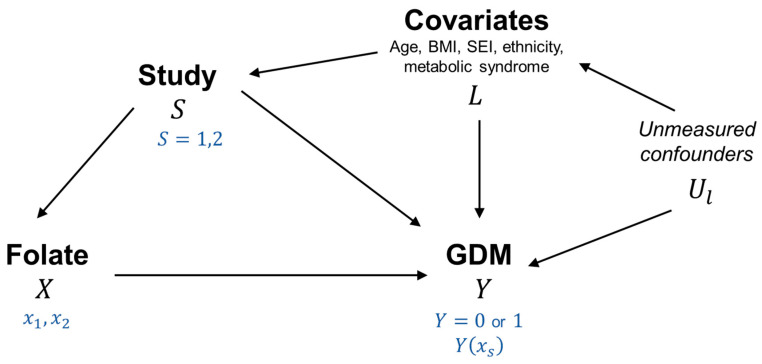
**Direct acyclic graph for the individual participant data meta-analysis**.

**Figure A2:**
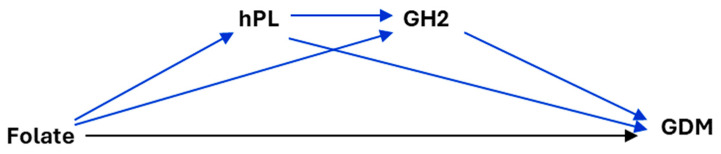
**Direct Acyclic Graph for mediation analysis of indirect (blue lines) and direct (black line) effects of maternal folate on GDM**.

## Data Availability

Data that support the findings of this study are openly available in Flinders University Repository of Open Access Data Sets (ROADS) at http://doi.org/10.25451/flinders.21721397 reference number 10.25451/flinders.21721397. All protocols and models related to this project are available upon reasonable written request to the corresponding author.

## Appendix A. IPTW Analysis Assumptions

The direct acyclic graph (DAG) for the individual participant data meta-analysis is as follows:

The following assumptions are made:

1. Consistency

The counterfactual outcome ($Y\left(x_{s}\right)$) agrees with the observed outcome Y for all individuals in study (S) exposed to folate (X). It is assumed that folate (X) is the only data generating mechanism for GDM (Y) and that the counterfactual outcome can be predicted from the observed X and Y.

2. Positivity

$$f\left(X=x|L\right)>0\;\mathrm{and}\;0<P\left(S_{i}=j|L_{i}\right)>1$$

There is balanced confounding between folate exposure status, such that any individual with characteristics (L) has a positive probability of being included (i.e., eligible to participate) in the other study, and positive probability of exposure to folate.

3. Ignorable treatment

$$Y\left(x_{s}\right)⊥X|L$$

Within each study, folate exposure (X) status is independent of the counterfactual outcome ($Y\left(x_{s}\right)$), conditioning on covariates (L). This assumes that individuals within a study with the same characteristics (L) would have the same outcome risk if given the same folate exposure.

4. Ignorable study alignment

$$Y\left(x_{s}\right)⊥S|L$$

Study indicator (S) is independent of the counterfactual outcome ($Y\left(x_{s}\right)$), conditioning on covariates (L). This assumes that individuals in different studies with the same characteristics (L) would have the same outcome risk if given the same folate exposure.

## Appendix B. Direct Acyclic Graph (DAG) for Mediation Analysis

Direct effect of maternal red cell folate on GDM is shown with black arrow. Blue arrows indicate the joint indirect effect of folate on GDM through hPL and GH2 (Folate → hPL→ GDM; folate → GH2 → GDM; folate → hPL → GH2 → GDM).
